# Supplementary material for: Use of Cardio-Pulmonary Ultrasound in the Neonatal Intensive Care Unit
Source: Children (Basel). 2023 Feb 26;10(3):462. doi: 10.3390/children10030462 (PMC10047372; doi:10.3390/children10030462)
Supplement: Supplementary file 1 [file children-10-00462-s001.zip › children-2169648-supplementary.pdf]

children

MDPI

Review

Use of cardio-pulmonary ultrasound in the Neonatal Intensive Care Unit

Elena Ciarmoli [1], Enrico Storti [2], Jessica Cangemi [3], Arianna Leone [3], and Maria Pierro [3,*]

1. Neonatal and pediatric unit, ASST Brianza, P.O. Vimercate, Vimercate, Italy

2 Department of Critical Care, Maggiore Hospital, Cremona, Cremona, Italy

3. Neonatal and Pediatric intensive care unit, Bufalini Hospital, Cesana AUSL Romagna, Italy

*Correspondence: Maria Pierro

maria.pierro93gmail.com; maria.pierro@auslromagna.it

Neonatal and Paediatric Intensive Care Unit

M. Bufalini Hospital,AUSL Romagna

Viale G. Ghirotti, 286 - 47521, Cesena, Italy

Phone number: +39/0547352844

SUPPLEMENTARY MATERIAL

1. Targeted neonatal ecochardiography

Targeted neonatal ecochardiography (TnECHO) performed by neonatologist has reached widespread use in Neonatal Intensive Care Units (NICU), allowing a real-time assessment of the cardiovascular state supporting clinical decision-making [1].

1.1 Patent ducts Arteriosus

In this section we provide a brief summary of the patent ductus arteriosus (PDA) measurements, referring Table1s for cut-off values of Hemodynamic significant PDA (HsPDA).

PDA evaluation criteria		Cut off Values		
		Small shunt	Medium shunt	Large shunt
**Ductal characteristics**				
-	PDA diameter (mm)	<1.5	1.5- 2	>2 or >1.4/kg
-	PDA/LPA ratio	<0.5	0.5-1	>1
-	Sistolic/diastolic velocity ratio	<2	2-4	>4
**Pulmonary overflow**				

-	LA/Ao ratio or LVEDD (mm)	<1.5	1.5-2	>2
-	LVO (ml/kg/min)	<200	200-300	>300
-	End-diastolic LPA velocity	<0.2	0.2-0.5	>0.5
	(mm/s)	<0.3	0.3-0.5	>0.5
-	Pulmonary vein diastolic wave velocity (mm/s)	>40	30-40	<30
**Systemic hypoperfusion**				
-	Medium/anterior cerebral artery	Forward	Forward	Absent/Reversed
	diastolic flow	Forward	Absent	Reversed
-	Truncus celiacus diastolic flow	Forward	Absent	Reversed
-	Descending aorta diastolic flow			

Table S1. Usual cut off values indicative of Hemodynamic significant Patent ducts Arteriosus (HsPDA)

LPA, left pulmonary artery; Ao, aorta; LVEDD, left ventricular end diastolic-diameter; IVRT, isovolumetric relaxation time; E/A early diastolic phase/late atrial contraction ratio

1.1.1. *Ductal dimension and characteristics*

Transductal patency and diameter (Figure S1A) are detectable from many views (except the four chamber wiew), although they are preferably measured from the left high-sided parasternal one ("the ductal view") at PDA narrowest point (usually at the pulmonary end). The PDA diameter may be expressed as an absolute value or standardized for patient's weight or left pulmonary artery (LPA) diameter.

Doppler gives important information about the speed of the PDA shunt in systole and diastole, enabling, in some cases, a reliable prediction about ductal closure. In particular, shunt could go from a restrictive shunt characterized by high systolic velocity peak and low systolic/diastolic gradient and likely to close on its own (Figure S1B) to a pulsatile, non-restrictive pattern, characterized by low peak systolic velocity and high systolic/diastolic gradient and unlikely to close on its own (Figure S1C) [2].

1.1.1. *Signs of pulmonary overflow*

The echocardiographic signs of pulmonary overflow are due to significant left to right ductal shunt that leads to increased pulmonary venous return which in turn causes left atrial volume overload and potentially subsequent left ventricular dysfunction.

Among these, left atrium to aortic root (LA/Ao) is measured from parasternal long axis view in M-mode, placing the cursor in correspondence of the aortic valve directed perpendicularly to the aorta (Figure S1D): the aortic valve diameter should be measured in telediastole, just before its opening, and the left atrium should be estimated in the frame in which its volume is maximum (during systolic phase) [3].

This view also allows the estimate of Left Ventricular End-Diastolic Diameter (LVEDD), with the difference that the cursor must be placed perpendicular to the interventricular septum, passing the mitral valve tip [4].

To correctly interpret these 2 measures is important to take into account the presence and the carrying capacity of shunt through the atrial defects, that could lead to underestimate the amount of pulmonary overflow.

LPA diastolic velocity is detected from high parasternal short axis is another parameter of ductal hemodynamic significance (Figure S1E) [4].

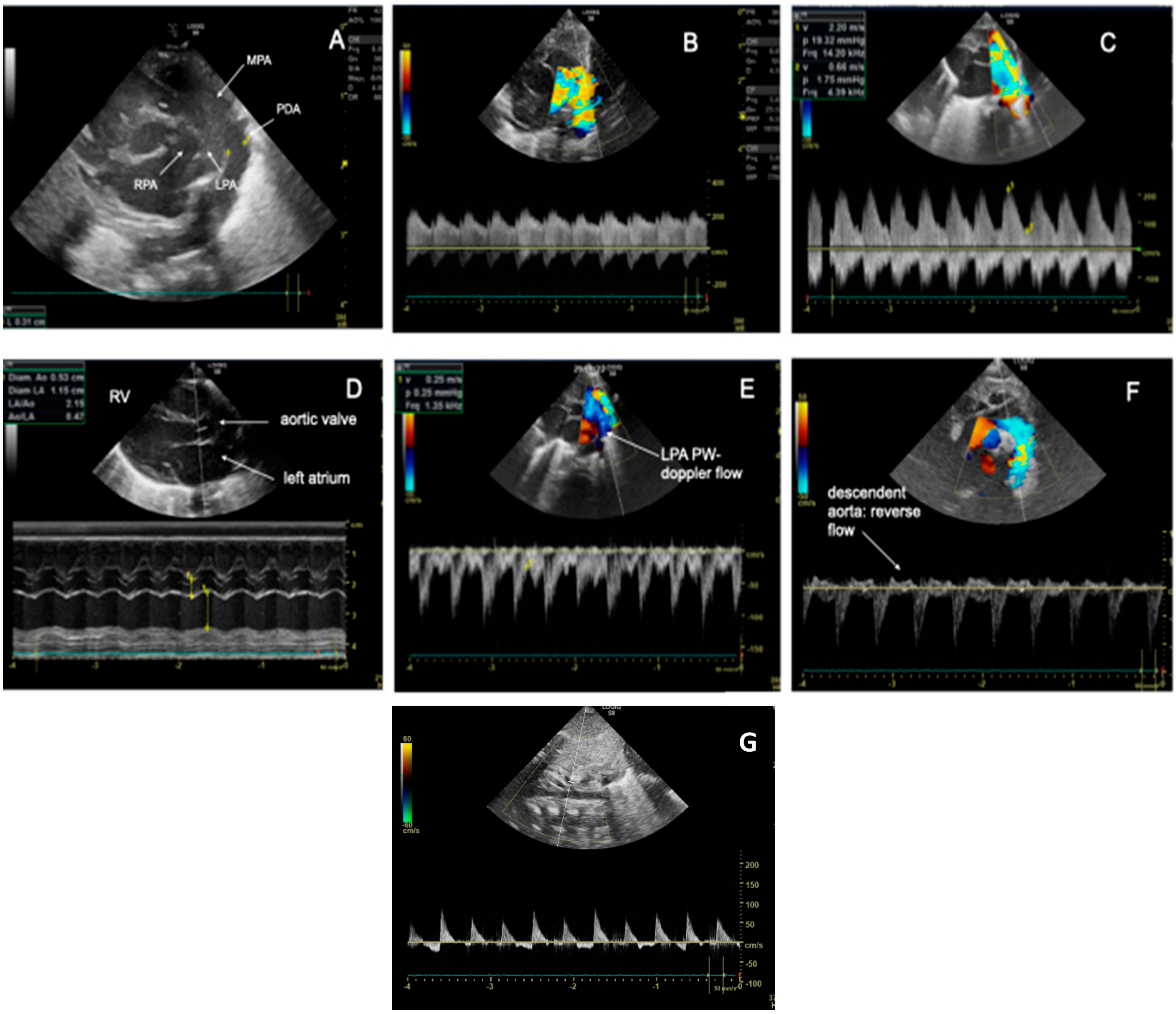

Figure S1. Hemodynamic assessment of the PDA
A) 2D image, left high-sided parasternal view (“ductal view”): study of transductal patency and diameter. B-C) color-Doppler, left high-sided parasternal view. B) left to right restrictive shunt characterized by high systolic velocity peak and low systolic/diastolic gradient. C) non-restrictive left to right shunt, characterized by low peak systolic velocity and high systolic/diastolic gradient. D) 2D parasternal long axis view, M-mode. Left atrium to aortic root ratio (LA/Ao) is measured placing the cursor in correspondence to the aortic valve directed perpendicularly to the aorta. LA/Ao > 1,5 is considered an indirect sign of pulmonary overflow. E) Pulse-wave doppler parasternal short axis view. Left pulmonary artery (LPA) diastolic velocity is measured placing the cursor over the left pulmonary artery. An increased end diastolic velocity (>0,2 m/s) is a marker pulmonary overflow., aortic arch view, Pulse-wave doppler. F) Absent or reversed diastolic flow in post-ductal descendent aorta is generally considered as a marker of systemic hypoperfusion and consequent diastolic organic ischemia. G) Similar evaluation can be done studying diastolic flow in the celiac truncus and superior mesenteric artery.

1.1.3. *Signs of systemic hypoperfusion*

Post-ductally stealing phenomena, such as absent or reversed diastolic flow indicative of systemic hypoperfusion must be evaluated. The districts tested with this purpose are the

truncus celiacus, superior mesenteric artery or descendent aorta from the subcostal sagittal view (Figure S1G).

1.2 Early and late Pulmonary hypertension (PH)

Some of the echographic parameters that describe these features are:

- the position of the interventricular septum (IVS) during the cardiac cycle, detected from the parasternal short axis view, can be used as an indirect indicator of elevated pulmonary resistance and systolic right ventricle (RV) pressure with different grades of severity according to its shape: a flat or D-shape suggests increased pulmonary pressures (Figure S2A). Another indirect sign of pulmonary hypertension is represented by the eccentricity index, derived from the ratio between respectively the parallel and perpendicular axis to the IVS. The normal value is=1, a a systolic ratio >1.15 is indicative of pressure overload to the RV .[5]

- the pattern of the flow across the pulmonary artery detected from doppler mode is informative about pressure gradient, as reflected by acceleration time/ejection time (AT/ET). Values below 0.3 suggest increased pulmonary pressures (Figure S2B). While studying the pulmonary artery pattern of flow, a "notch" (Figure S2D) could be detected indicative or high pulmonary vascular resistance (PVR) [5] .

- trans-tricuspidalic jet (Figure S2C), that provides an estimate of the pulmonary artery systolic pressure (PASP), it is detectable from many views, but best measured from the 4chamber view placing continuous doppler just over the tricuspid valve plane. This allows the measurement of the peak velocity of blood flow between the two chambers, in turn converted into a pressure gradient by applying the modified Bernoulli equation: pressure (mmHg) = 4 x velocity. Values ≥2.5 m/sec are considered markers of high systolic PAP but its absence does not exclude the presence of PH. Likewise, the measure of the pulmonary regurgitation jet across the pulmonary vein is indicative of medium PAP, with normal values<2.2 m/sec [5];

- RtL intra-cardiac shunts and/or at the level of the PDA suggest PH, as long as congenital heart diseases has been excluded[2] (Figure S2F).

Table S2 and Figure S2 summarize the most important TnECHO parameters used in the evaluation fo PH in the neonatal peri

**PPHN evaluation criteria**	**CUT OFF VALUES**
**Severity of the pulmonary hypertension**	
- Derived PASP	<2.5 m/sec
**Right ventricular performance and pulmonary hemodynamics**	
- IVS flattening	O-shape LV at end systole: PAPs<50%PAS<br>flat shape at end-systole: PAPs 50-100%P<br>D-shape LV at end systole: PAPs>50%PAS
- EI	>1.15
- AT/ET	<1.15
- TAPSE	ELBW <4-5 mm - term newborns < 9-11 mm
**Left ventricular performance and systemic blood flow**	
- FS (%)	<28%
- EF %	<41-50%

Table S2. Persistent pulmonary hypertension of the newborn (PPHN) at Targeted neonatal echocardiography (TnECHO) evaluation. PAPS, pulmonary artery systolic pressure; IVS, interventricular septum; EI, eccentricity index; AT/ET, acceleration time/ejection time ratio; TAPSE, tricuspid annular plane systolic excursion.

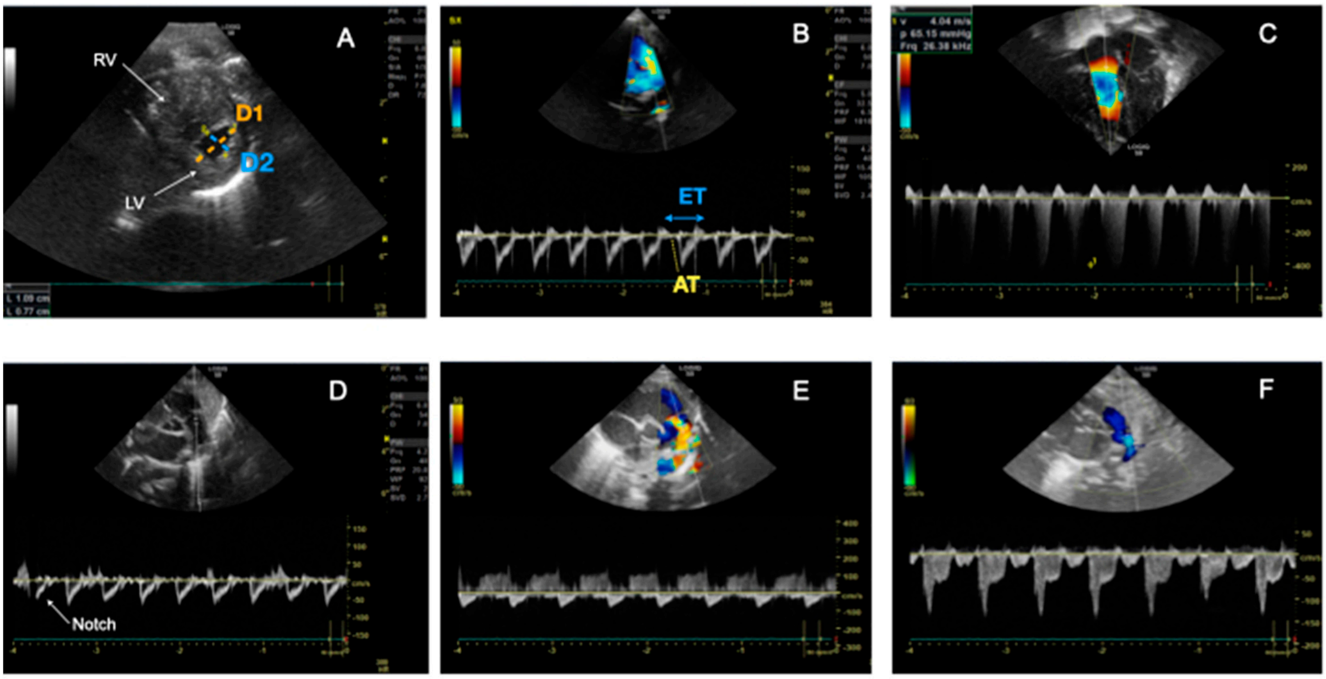

Figure S2. Indirect signs of pulmonary hypertension (PH)

A) Eccentricity index (EI) in short axis view. B) acceleration time/ejection time (AT/ET) in short axis view, color-Doppler mode.: acceleration time/ejection time across pulmonary artery. C) transtricuspidalic gradient in color-Dopler mode, four chamber view. D) pulmonary artery "notch" short axis view, PW- Doppler. E) Patent ductus arteriosus (PDA) bidirectional pattern, with right to left shunt > 30% of the cardiac cycle. F) complete right to left shunt, indicative of severe pulmonary hypertension.

1.3 Echocardiographic signs of hypovolemia

Hypovolemia is detected at TnECHO, combining multiple aspects, such as reduced left ventricular end-diastolic diameter, evidence of "kissing walls" (collapse of the left ventricular walls at end-systole to the intra-ventricular septum), reduced hepatic venous flow and collapsed vena cava [6] (Figure S3)

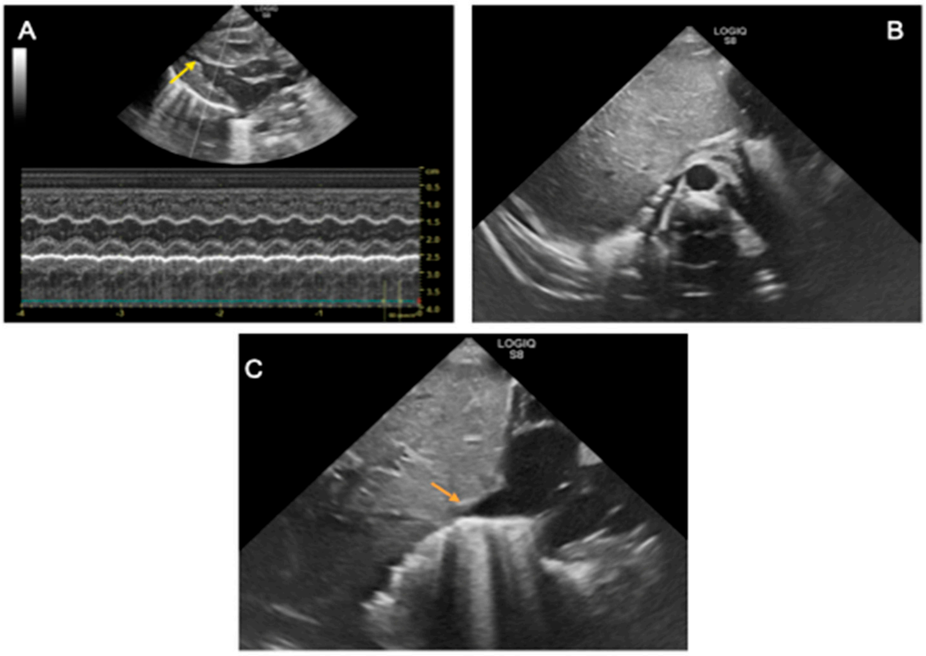

Figure S3. Signs of hypovolemia

A) "Kissing" of the left ventricle and interventrcular septum (yellow arrow) in Lon-axis parasternal view B) reduced representation of the hepatic vessels; C) collapse of the inferior vena cava (orange arrow)

1.4 Echocardiographic signs of ventricular dysfunction

1.4.1 Left ventricle dysfunction

- Decreased fractional shortening (FS%): FS is the reduction of the length of the end-diastolic diameter that occurs by the end of systole. Values below 28% are abnormal [7] (Figure S3A).

- Decreased ejection fraction (EF%) EJ is the volumetric fraction of blood ejected from the left ventricle with during systole in relation to the total end-diastolic volume. A decreased EF below 35% is a sign of ventricular dysfunction [7] (Figure S3B,C)

1.4.2 Right ventricle dysfunction

- Tricuspid annular plane systolic excursion (TAPSE) is a parameter of global RV function which describes apex-to-base shortening. Reduced TAPSE is a sign of right ventricular dysfunction. The normal ranges for TASE are gestational age dependent [7] (table

- Right ventricular fractional area change (RVFAC) provides an estimate of the global RV systolic function. The formula to calculate the percentage of area change within the RV between diastole and systole. A decreased RVFAC below 35% is considered abnormal [7]l (Figure S3D,E)

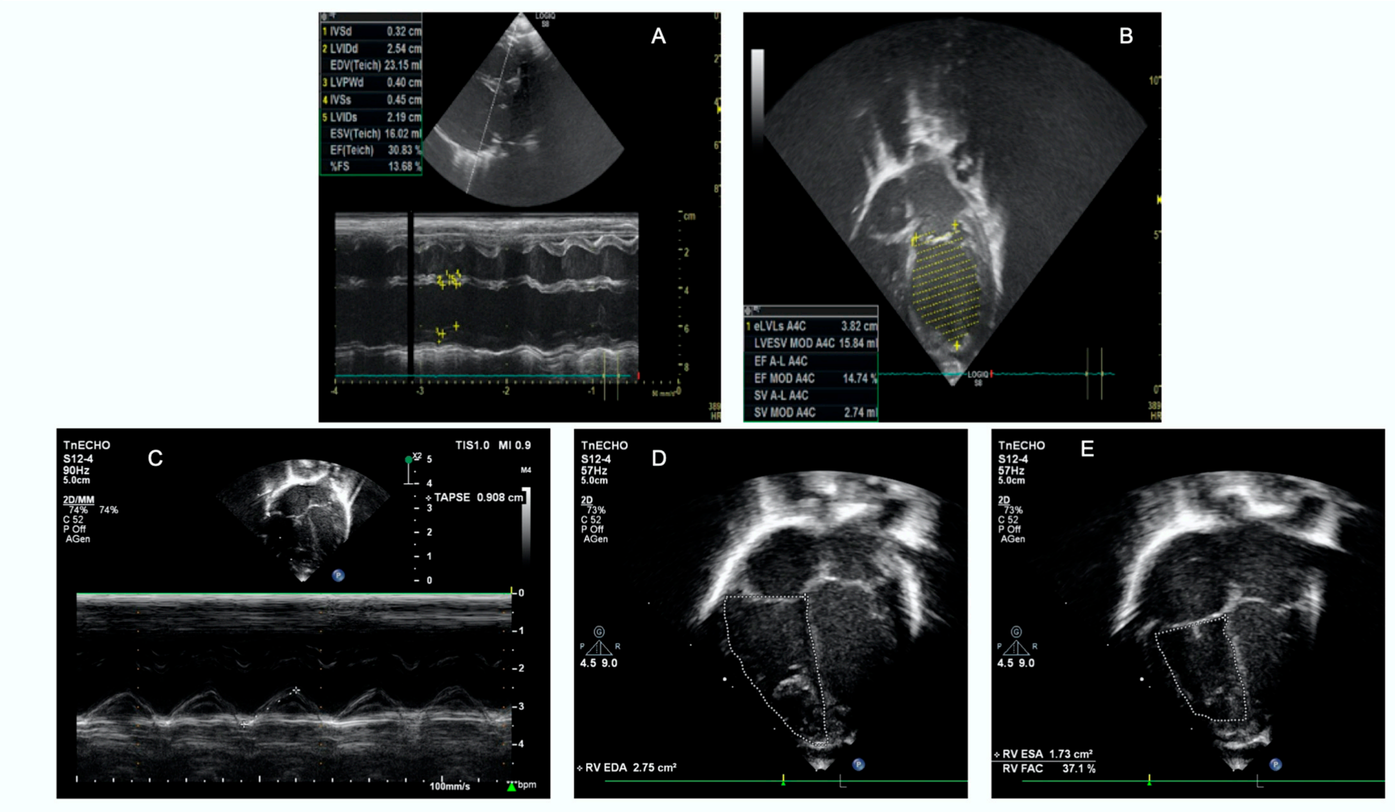

Figure S4. Right and left ventricular function
A) Parasternal long axis view at the level of the papillary muscles, 2D M-mode. Evaluation of left ventricular (LV) diameters and thickness, showing significantly decreased shortening fraction (SF%) and ejection fraction (EF). B) four chamber view, Simpson function. Evaluation of left ventricle LV function, showing significantly decreased EF
C) four chamber view, 2D M-mode at the level of the external part of the tricuspid valve tricuspid. Right ventricular (RV) function evaluation using Tricuspid annular plane systolic excursion (TAPSE), showing mild right ventricular dysfunction D-E), four chamber view. RV function evaluation using FAC (fractional area change), showing mild right ventricular dysfunction

Figure S3. Cardiac function
A) Parasternal long axis view at the level of the papillary muscles, 2D M-mode. Evaluation of left ventricular (LV) diameters and thickness, showing significantly decreased shortening fraction (SF%) and ejection fraction (EF). B) four chamber view, Simpson function. Evaluation of left ventricle LV function, showing significantly decreased EF
C) four chamber view, 2D M-mode at the level of the external part of the tricuspid valve tricuspid. Right ventricular (RV) function evaluation using Tricuspid annular plane systolic excursion (TAPSE), showing mild right ventricular dysfunction D-E), four chamber view. RV function evaluation using FAC (fractional area change), showing mild right ventricular dysfunction
Measurements of left ventricular systolic performance. A) Shortening Fraction (SF) using M-mode**;** **B**) Ejection Fraction (EF) derived from 4chamber view with Simpson's Biplane rule

3. Lung Ultrasound (LUS)

3.2 Detection of lung diseases

LUS provides important information guiding differential diagnosis between many pathological processes involving the lung.

3.4.1 Transient tachypnea of the newborn (TTN)

TTN or "wet lung" is a condition of respiratory distress characterized by delayed lung fluid clearance. LUS examination reveals a normal pleural line, with variable presence of non compact B-lines involving one or both lung. [8] A possible image in TTN is the "double lung point" (Figure S5A), which is the presence of a clear discrepancy between superior and inferior pulmonary fields in terms of density of B-line [9]. In severe forms of TTN there is a diffuse B2 pattern and the double lung point could manifest later, after improvement of pathological process.

3.2.2 Respiratory distress Syndrome (RDS)

RDS is a homogeneous, and restrictive lung disease of the premature due to primary surfactant deficiency. LUS can show thickened and irregular pleural line with a white lung image. All the lung's districts are involved [10]. This pattern is usually accompanied by the ground-glass opacity (GOS) sign, characterized by mild, regularly distributed lung consolidation with no obvious air bronchograms (Figure S5B), or by the snowflake (SFS) sign characterized by regularly distributed lung consolidation with air bronchogram that resembles a snow pattern [8, 11] The degree and extent of snow flake consolidations is associated with the severity of the disease [12].

3.4.3 Meconium aspiration syndrome

Meconium aspiration syndrome (MAS) is an obstructive inhomogeneous neonatal lung disease with variable degree of severity, affecting mainly term and post term newborn and induced by inhalation of meconium-stained amniotic fluids during labor and delivery [13]. This condition is characterized by a variable degree of hypoxia, acidemia, inflammation, airway obstruction and potentially surfactant dysfunction [13] At LUS exam, MAS appears as an inhomogeneous pattern, with subpleural consolidation associated with pleural B-line and spared areas (Figure S5D*) [14]*.

3.4.5 Pulmonary malformations and congenital diaphragmatic hernia

Congenital diaphragmatic hernia (CDH) is a life-threatening condition characterized by variable presence of intestinal viscera (stomach, liver, intestine) in the thoracic cavity secondary to a wall defect in the diaphragm [15]. It could be studied with LUS to point out the presence of parenchymal organs or bowel loops in the chest replacing normal lung sliding or to corroborate absence of pleural line or absent diaphragm on the affected side (Figure S5D) [16].

Congenital pulmonary airway malformation will appear with LUS as cystic lesions without pleural line and lung consolidation in the affected side.

3.4.6 Bronchopulmonary dysplasia

BPD, the chronic lung disease of prematurity, is defined as O2-dependency at 36week post-gestational age [17].

BPD comprises different phenotypes, including: parenchimal disease, airway vasoreactivicy and anatomical constriction, pulmonary vascular disease, and involvement of the interstitial, even. LUS show various findings, such as inhomogeneous subpleural consolidations, irregular pleural line, co-presence of areas with regular appearance or compact B-lines, or white lung [18].

Distribution of B-lines may help assess interstitial space, distinguishing from a congestive phenotype from inflammatory interstitial disorder (ILD). ILD is characterized by irregular pleural line and irregular distribution of the B-lines *(BPD-* Figure S5E*)*, while the congestive phenotype is characterized by a more regular distribution of the B-lines with a gravity dependent pattern [19] *(BPD-* Figure S5F*)*.

3.4.7 Neonatal acute respiratory distress syndrome

Neonatal acute respiratory distress syndrome was first defined by De Luca an colleuages [20]

There are no specific LUS criteria in neonatal acute respiratory distress syndrome. In adults LUS reliably identifies ARDS morphology: non-uniformly distributed B-lines, lung atelectasis, pleural lines irregularities [21]. Similar findings can be found in neonates with clinically diagnosed NARDS (Figure S4G)

3.4.4 Pneumonia

Pneumonia appears in LUS as lung consolidation with dynamic livel-like air or fluid bronchogram. Lung sliding disappears, pleural line may also appear irregular with disruption and coarse appearance [22]. Characteristic are signs of air trapping in patent bronchi, seen as branching linear echogenicities that move with respiration, differently from atelectasis [22]. If fluid or mucous material accumulates bronchogram appears, showing hypoechoic branching structures. If consolidation is close enough to the heart "lung pulse" could be detected (evidence that consolidation pulses synchronous with the heart beat) [22]. Pleural effusion can be present as well (Figure S4H)

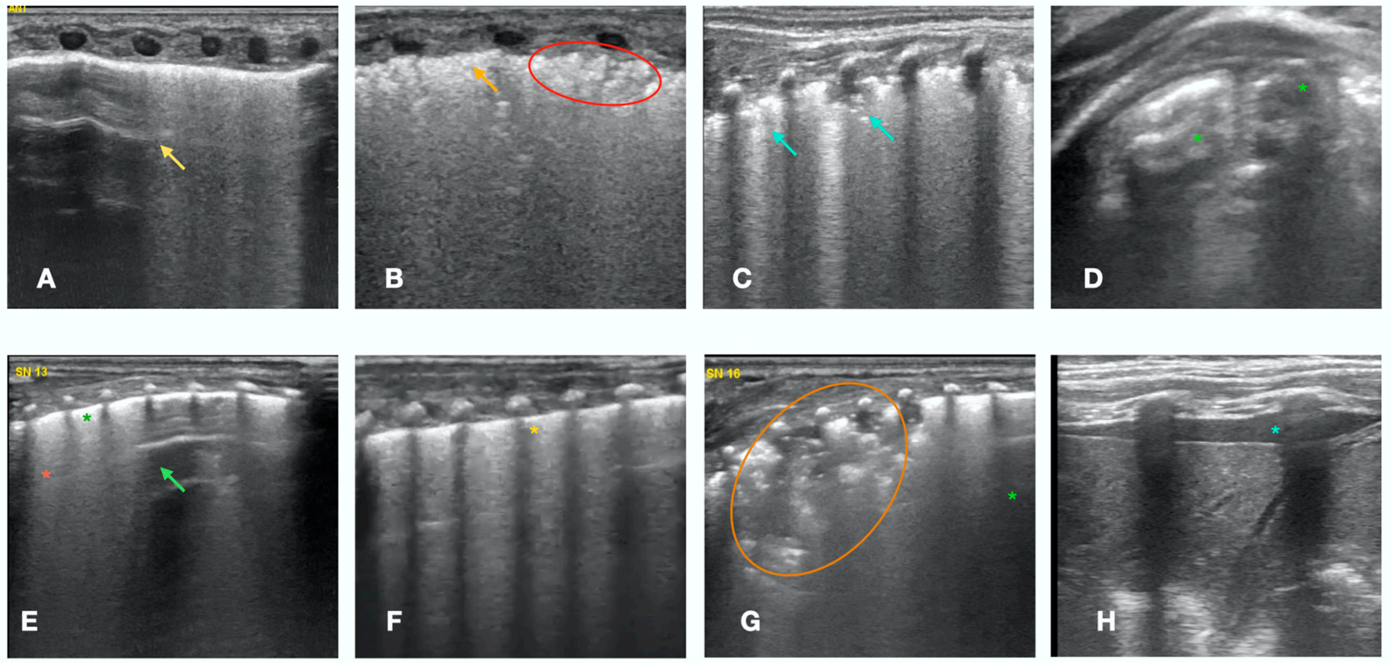

Figure S5. Examples of Lung diseases detected at Lung Ultrasound (LUS).
A) transient tachypnea of the newborn (TTN) or "wet lung", with "double lung point" (yellow arrow) B) respiratory distress syndrome (RDS) with thickened pleural line (orange arrow) and ground-glass opacity sign (GOS) (red circle); C) Meconium aspiration syndrome (MAS) with irregular atelectasis (light-blue arrows); D) Congenital diaphragmatic hernia (CDH) with intestinal loops in the thorax (green asterisks); E) Interstitial lung disease (ILD) associated with Bronchopulmonary dysplasia (BPD) characterized by irregularly distributed coalescent B-lines (red asterisk), spared areas (green arrow) and thickened pleural line (green asterisk), suggesting an inflammatory interstitial involvement; F) Evolving BPD with lung congestion characterized by regularly distributed and gravity depend B-lines with regular pleural line (yellow asterisk), suggesting pulmonary oedema; G) Neonatal acute respiratory distress syndrome (NARDS) with areas of lung derecruitment (orange circle) an spared areas (green asterisk); H) Pneumonia with lung derecruitment and a small pleural effusion (light-blue asterisk)

References

1. El-Khuffash, A.; Herbozo, C.; Jain, A.; Lapointe, A.; McNamara, P. Targeted neonatal echocardiography (TnECHO) service in a Canadian neonatal intensive care unit: a 4-year experience. *J. Perinatol.* **2013**, *33*, 687–690, https://doi.org/10.1038/jp.2013.42.
2. van Laere, D.; van Overmeire, B.; Gupta, S.; El-Khuffash, A.; Savoia, M.; McNamara, P.J.; Schwarz, C.E.; de Boode, W.P. Application of NPE in the assessment of a patent ductus arteriosus. *Pediatr. Res.* **2018**, *84* (Suppl. S1), 46–56. https://doi.org/10.1038/s41390-018-0077-x.
3. Arlettaz, R. Echocardiographic Evaluation of Patent Ductus Arteriosus in Preterm Infants. *Front. Pediatr.* **2017**, *5*, 147. https://doi.org/10.3389/fped.2017.00147.
4. Singh, Y.; Fraisse, A.; Erdeve, O.; Atasay, B. Echocardiographic Diagnosis and Hemodynamic Evaluation of Patent Ductus Arteriosus in Extremely Low Gestational Age Newborn (ELGAN) Infants. *Front. Pediatr.* **2020**, *8*, 573627. https://doi.org/10.3389/fped.2020.573627.
5. More, K.; Soni, R.; Gupta, S. The role of bedside functional echocardiography in the assessment and management of pulmonary hypertension. *Semin. Fetal Neonatal Med.* **2022**, *27*, 101366. https://doi.org/10.1016/j.siny.2022.101366.
6. Fathi, E.M.; Narchi, H.; Chedid, F. Noninvasive hemodynamic monitoring of septic shock in children. *World J. Methodol.* **2018**, *8*, 1–8, https://doi.org/10.5662/wjm.v8.i1.1.
7. Levy, P.T.; Tissot, C.; Horsberg Eriksen, B.; Nestaas, E.; Rogerson, S.; McNamara, P.J.; El-Khuffash, A.; de Boode, W.P. Application of Neonatologist Performed Echocardiography in the Assessment and Management of Neonatal Heart Failure unrelated to Congenital Heart Disease. *Pediatr. Res.* **2018**, *84* (Suppl. S1), 78–88. https://doi.org/10.1038/s41390-018-0075-z.
8. Liu, J.; Wang, Y.; Fu, W.; Yang, C.-S.; Huang, J.-J. Diagnosis of Neonatal Transient Tachypnea and Its Differentiation From Respiratory Distress Syndrome Using Lung Ultrasound. *Medicine* **2014**, *93*, e197, https://doi.org/10.1097/md.0000000000000197.
9. He, L.; Sun, Y.; Sheng, W.; Yao, Q. Diagnostic performance of lung ultrasound for transient tachypnea of the newborn: A meta-analysis. *PLoS ONE* **2021**, *16*, e0248827. https://doi.org/10.1371/journal.pone.0248827.
10. Copetti, R.; Cattarossi, L.; Macagno, F.; Violino, M.; Furlan, R. Lung ultrasound in respiratory distress syndrome: A useful tool for early diagnosis. *Neonatology* **2008**, *94*, 52–59. https://doi.org/10.1159/000113059.
11. Liu, J.; Fu, W.; Qin, S.-J. Lung ultrasound to guide the administration of exogenous pulmonary surfactant in respiratory distress syndrome of newborn infants: A retrospective investigation study. *Front. Pediatr.* **2022**, *10*, 1768. https://doi.org/10.3389/fped.2022.952315.
12. Guo, B.B.; Pang, L.; Yang, B.; Zhang, C.; Chen, X.Y.; OuYang, J.B.; Wu, C.J. Lung Ultrasound for the Diagnosis and Management of Neonatal Respiratory Distress Syndrome: A Minireview. *Front. Pediatr.* **2022**, *10*, 864911. https://doi.org/10.3389/fped.2022.864911.
13. Olicker, A.; Raffay, T.; Ryan, R. Neonatal Respiratory Distress Secondary to Meconium Aspiration Syndrome. *Children* **2021**, *8*, 246, https://doi.org/10.3390/children8030246.
14. Liu, J.; Cao, H.Y.; Fu, W. Lung ultrasonography to diagnose meconium aspiration syndrome of the newborn. *J. Int. Med. Res.* **2016**, *44*, 1534–1542. https://doi.org/10.1177/0300060516663954
15. Zani, A.; Chung, W.K.; Deprest, J.; Harting, M.T.; Jancelewicz, T.; Kunisaki, S.M.; Patel, N.; Antounians, L.; Puligandla, P.S.; Keijzer, R. Congenital diaphragmatic hernia. *Nat. Rev. Dis. Prim.* **2022**, *8*, 37. https://doi.org/10.1038/s41572-022-00362-w
16. Corsini, I.; Parri, N.; Coviello, C.; Leonardi, V.; Dani, C. Lung ultrasound findings in congenital diaphragmatic hernia. *Eur. J. Pediatr.* **2019**, *178*, 491–495, https://doi.org/10.1007/s00431-019-03321-y.
17. Higgins, R.D.; Jobe, A.H.; Koso-Thomas, M.; Bancalari, E.; Viscardi, R.M.; Hartert, T.V.; Ryan, R.M.; Kallapur, S.G.; Steinhorn, R.H.; Konduri, G.G.; et al. Bronchopulmonary Dysplasia: Executive Summary of a Workshop. *J. Pediatr.* **2018**, *197*, 300–308, https://doi.org/10.1016/j.jpeds.2018.01.043.
18. Aldecoa-Bilbao, V.; Velilla, M.; Teresa-Palacio, M.; Esponera, C.B.; Barbero, A.H.; Sin-Soler, M.; Sanz, M.I.; Roigés, M.D.S. Lung Ultrasound in Bronchopulmonary Dysplasia: Patterns and Predictors in Very Preterm Infants. *Neonatology* **2021**, *118*, 537–545, https://doi.org/10.1159/000517585.
19. Bruno, G.; Chioma, R.; Storti, E.; De Luca, G.; Fantinato, M.; Antonazzo, P.; Pierro, M. Targeted management of evolving and established chronic lung disease of prematurity assisted by cardiopulmonary ultrasound: A case report of four patients. *Front. Pediatr.* **2023**, *10*, 1112313. https://doi.org/10.3389/fped.2022.1112313.
20. De Luca, D.; van Kaam, A.H.; Tingay, D.G.; Courtney, S.E.; Danhaive, O.; Carnielli, V.P.; Zimmermann, L.J.; Kneyber, M.C.J.; Tissieres, P.; Brierley, J.; et al. The Montreux definition of neonatal ARDS: Biological and clinical background behind the description of a new entity. *Lancet. Respir. Med.* **2017**, *5*, 657–666. https://doi.org/10.1016/s2213-2600(17)30214-x.
21. Baston, C.; West, T.E. Lung ultrasound in acute respiratory distress syndrome and beyond. *J. Thorac. Dis.* **2016**, *8*, E1763–E1766, https://doi.org/10.21037/jtd.2016.12.74.
22. Liu, J.; Liu, F.; Liu, Y.; Wang, H.-W.; Feng, Z.-C. Lung Ultrasonography for the Diagnosis of Severe Neonatal Pneumonia. *Chest* **2014**, *146*, 383–388, https://doi.org/10.1378/chest.13-2852.

Disclaimer/Publisher's Note: The statements, opinions and data contained in all publications are solely those of the individual author(s) and contributor(s) and not of MDPI and/or the editor(s). MDPI and/or the editor(s) disclaim responsibility for any injury to people or property resulting from any ideas, methods, instructions or products referred to in the content.
